# Association Between Inflammation and Appetite in Healthy Community-Dwelling Older Adults—An *enable* Study

**DOI:** 10.3389/fragi.2022.826816

**Published:** 2022-03-18

**Authors:** Neshat Chareh, Eva Kiesswetter, Robert Kob, Anne Hannink, Beate Brandl, Thomas Skurk, Hans Hauner, Cornel C. Sieber, Dorothee Volkert

**Affiliations:** ^1^ Institute for Biomedicine of Aging, Friedrich-Alexander-Universität Erlangen-Nürnberg, Nuremberg, Germany; ^2^ ZIEL–Institute for Food and Health, Technical University of Munich, Freising, Germany; ^3^ Institute of Nutritional Medicine, Else Kroener-Fresenius-Centre for Nutritional Medicine, School of Medicine, Technical University of Munich, Munich, Germany; ^4^ Department of Medicine, Kantonsspital Winterthur, Winterthur, Switzerland

**Keywords:** inflammation, C-reactive protein, appetite, community-dwelling, aged

## Abstract

Aging is associated with reduced appetite as well as a slight increase in pro-inflammatory status, which both might contribute to the development of malnutrition. We aimed to evaluate the association between inflammation based on serum C-reactive protein (CRP), and appetite in healthy community-dwelling older adults. In this cross-sectional study of 158 healthy and non-smoking persons (aged 75–85 years), appetite was assessed in personal interviews by a single question with five answer categories. As nobody reported (very) poor appetite, the remaining three categories were dichotomised into “(very) good” and “moderate” appetite. Fasting serum CRP was analysed according to standard procedures, values ≥ 5.0 mg/L were considered as inflammation. The association between inflammation and appetite was examined by binary logistic regression, unadjusted and adjusted for age, sex, waist circumference, leptin, depressive mood, number of medications, interleukin-6 and tumor necrosis factor-α. Appetite was very good in 27.8%, good in 58.9%, and moderate in 13.3% of participants. Inflammation was present in 10.8% overall, in 8.8% of those with (very) good and in 23.8% of those with moderate appetite (*p* = 0.038). In the unadjusted model, participants with inflammation were 3.2 times more likely to have moderate appetite (95%CI: 1.01–10.44, *p* = 0.047). In the adjusted model, the odds of having moderate appetite was 3.7 times higher in participants with inflammation, but no longer significant (95%CI: 0.77–18.55, *p* = 0.102). In healthy older people, we found hints for a potential association between increased levels of CRP and a slightly reduced appetite. More studies in larger samples are needed.

## Introduction

Aging is associated with multiple changes in appetite regulation, including physiological alteration in hormonal and neurotransmitter regulation of food intake, which is described as the anorexia of aging (Morley, 2002). In addition, changes in the social environment, use of medications, chronic and acute diseases may further reduce appetite in older adults (Malafarina et al., 2013; Cox et al., 2020). A poor appetite is experienced by 10–22% of community-dwelling older individuals (Lee et al., 2007; Schilp et al., 2011; van der Meij et al., 2017). It should be considered as a warning signal for the reduction of food and energy intake, leading to weight loss and malnutrition and subsequently increasing the risk of impaired health and reduced quality of life in older persons (Lee et al., 2007).

Another major characteristic of the aging process is a progressive increase in the pro-inflammatory status in the absence of overt infection (Franceschi et al., 2000). This phenomenon has been described as “inflammaging”, which is characterized by slightly increased circulating levels of inflammatory biomarkers such as C-reactive protein (CRP), interleukin-6 (IL-6), and tumor necrosis factor-alpha (TNF-α) (Ballou et al., 1996; Bruunsgaard et al., 2000; Franceschi et al., 2000; Forsey et al., 2003; Franceschi and Campisi, 2014). In previous studies in high functioning older adults (mean age >70 years) with at least 2 years of follow-up, higher levels of inflammation markers at baseline were associated with an increased risk of adverse consequences such as functional (Reuben et al., 2002) and cognitive decline (Yaffe et al., 2003), and increased risk of mortality (Harris et al., 1999; Reuben et al., 2002).

Reduced appetite in case of acute diseases and pronounced inflammation is well known from clinical practice, and an association between poor appetite and increased serum concentration of CRP has been reported recently in geriatric patients hospitalized in acute care ward (mean age >80 years) (Sieske et al., 2019). Also, an association between higher levels of TNF-α and impaired appetite was observed in well-functioning community-dwelling older adults (mean age 74 years) (Lee et al., 2007). The early detection of any potential contributing factors in deterioration of appetite before they lead to serious problems could help older adults maintain their health and consequently prevent unfavorable health issues, such as malnutrition. To the best of our knowledge, no further data regarding the association of inflammation and appetite in older persons without acute diseases is available. In the present study, we aimed to examine the association between CRP, as an inflammatory marker, and appetite in healthy community-dwelling older adults.

## Materials and Methods

### Study Design and Recruitment

This explorative analysis used cross-sectional data of the phenotyping platform “*enable*”, which is described in detail elsewhere (Brandl et al., 2020). Data were assessed between April 2016 and March 2018 in 159 community-dwelling adults aged 75–85 years, living in Freising (*n* = 54) or Nuremberg (*n* = 105), two cities in Southern Germany. Participants were healthy and able to come independently to the study centers. The general eligibility of participation was screened by a standardized telephone interview and, thereafter, potentially individuals were invited to the study center for a screening visit. Exclusion criteria were BMI (Body Mass Index) less than 18.5 or greater than 35 kg/m^2^, unintended weight loss of more than 5% in the previous 3 months, cognitive impairment (MMSE (Mini Mental State Examination) < 24 points), smoking, need of care (classification into a care level according to German Social Security Code), and recent participation in an intervention study. Moreover, participants with physician-diagnosed chronic diseases (self-reported/medication list) such as human immunodeficiency virus infection, liver disease, known diabetes mellitus, endocrine disease, autoimmune disease, lung disease, stomach ulcer, renal failure requiring dialysis, untreated hypertension, blood transfusion in the last 3 months, or the occurrence of heart failure, stroke, coronary heart disease, cancer, or psychological, neurological or neurodegenerative disease within the previous 3 years were excluded from the study. All data were assessed at two study visits within 1 week in the study centers (Brandl et al., 2020).

The study was conducted in line with the Declaration of Helsinki. The study protocol was approved by the ethical committee of the Faculty of Medicine of the Technical University of Munich (approval no. 452/15) and the Friedrich-Alexander-Universität Erlangen-Nürnberg (approval no. 291/15B). The study was registered at the German Clinical Trials Register (DRKS-ID: DRKS00009797). All participants signed the informed consent form prior to the start of the assessments.

### Participants’ Characteristics

Participants recorded their age, sex, living situation, and currently taken medications in standardized questionnaires. The emotional status was assessed using the Geriatric Depression Scale (GDS; 0–15 points), with a score of 5 points and below defining a non-depressive mood (Yesavage et al., 1982). Nutritional status was determined by the Mini Nutritional Assessment-Long Form (MNA-LF; 0–30 points) where participants with a score above 23.5 are considered to be well-nourished (Vellas et al., 1999). Both, GDS and MNA were conducted in personal interviews. Anthropometric measurements were performed in the morning following overnight fasting. Bodyweight was measured in light clothing to the nearest 0.5 kg (seca Medical Body Composition Analyser, mBCA 515, Seca GmbH & Co. KG, Hamburg, Germany), and body height was measured to the nearest 0.1 cm in a standing position without shoes using a stadiometer (Seca GmbH & Co. KG, Hamburg, Germany). BMI was calculated as weight (kg)/height (m^2^). Waist circumference was measured to the nearest 0.1 cm at the midpoint between the lowest rib and the iliac crest with a measuring tape (Seca GmbH & Co. KG, Hamburg, Germany). Physical performance was assessed by the Short Physical Performance Battery (SPPB; 0–12 points), which consists of three tests (standing balance, gait speed, and chair-rise) with an individual score of 0–4 points for each test (Guralnik et al., 1994). A score of at least 10 indicates no limitations (Guralnik et al., 1995). All measurements and assessments were performed according to standard procedures by trained staff.

### Appetite

Participants’ recorded their appetite by the self-reported question: “How would you roughly estimate your appetite?” with five answer categories (very good, good, moderate, poor, and very poor).

### Blood Biomarkers

Blood samples were obtained after overnight fasting. Serum CRP was measured by a laboratory service provider (Synlab Holding, Munich, Germany) using standard procedures. Levels below 5.0 mg/L were considered as no inflammation (= normal values), and levels of 5.0 mg/L and higher as inflammation (Dati et al., 1996). One participant with a CRP level above 100 mg/L was excluded from the present analyses due to potential acute infection (Clyne and Olshaker, 1999). The concentration of IL-6 and TNF-α (inflammation markers) and leptin (adipose-related marker) in EDTA-plasma (ethylenediaminetetraacetic acid- plasma) was determined by a Human Magnetic Luminex^®^ Assay (Catalog no.: LXSAHM, R&D Systems Europe Ltd., Abingdon, United Kingdom). The assay was performed according to the protocol provided by the manufacturer.

### Statistical Analyses

For the statistical analyses, those who reported a very good or good appetite were combined into one group as “(very) good appetite”, resulting in two appetite categories, “(very) good” and “moderate” since no one reported poor or very poor appetite. Categorical data are presented as percentage, continuous data as median and interquartile range (P25–P75) if they are non-normally distributed, and as mean and standard deviation (SD) if they are normally distributed. Participants’ characteristics and inflammation status were compared according to participants’ appetite by applying the chi-square test for categorical data and the Mann-Whitney-U-test or t-test for continuous data. The association between inflammation (CRP ≥ vs <5.0 mg/L) and appetite was evaluated by chi-square test, followed by binary logistic regression analysis with appetite ((very) good vs moderate) as the dependent variable. Four regression models were calculated, model 1 was unadjusted. In the three adjusted models, potential confounders were selected based on the literature (Yeh and Schuster, 1999; Morley, 2002; McDade et al., 2006; O’Connor et al., 2009; Cox et al., 2020; Aprahamian et al., 2021). Model 2 was adjusted for demographics variables (age and sex), model 3 was further adjusted for health variables (waist circumference, leptin, GDS score, and the number of medications), and in model 4 other inflammation markers (IL-6 and TNF- α) were added. A p-value <0.05 was considered as a statistically significant result. Statistical analysis was performed using the SPSS version 25.0 (IBM, Munich, Germany).

## Results

### Participants’ Characteristics

Participants’ median age was 77.0 years, 49.4% were female, and 54.4% lived alone. 27.8% reported very good, 58.9% good, and 13.3% moderate appetite. The median SPPB score was 12.0 (P25–P75: 11.0–12.0) in the total group and only 5.7% had limitations in physical performance. All except two participants showed no depressive mood, and none was malnourished according to MNA. Participants with moderate appetite had significantly higher median GDS score and significantly lower MNA score compared to those with a (very) good appetite. Participants with (very) good and moderate appetite did not differ with respect to SPPB score, number of medications, BMI, waist circumference, leptin and, levels of IL-6 and TNF-α (Table 1).

**TABLE 1:** Participants’ characteristics in the total sample size according to appetite.

		Total (*n* = 158)	(Very) Good appetite (*n* = 137)	Moderate appetite (*n* = 21)	*p* value
Age [years], median (P25–P75)		77.0 (76.0–80.0)	77.0 (76.0–80.0)	78.0 (76.0–82.0)	0.168
Living situation: alone, n (%)		86 (54.4%)	71 (51.8%)	15 (71.4%)	0.093
Sex: Female, n (%)		78 (49.4%)	65 (47.4%)	13 (61.9%)	0.217
Medications, median (P25–P75)		2.0 (1.0–4.0)	2.0 (1.0–4.0)	3.0 (1.0–4.5)	0.167
GDS, median (P25–P75)		1.0 (0.0–2.0)	1.0 (0.0–1.0)	2.0 (1.0–4.0)	0.001
MNA-LF, Median (P25–P75)		27.5 (25.5–28.5)	27.5 (26.0–28.5)	26.5 (23.7–27.5)	0.007
Malnutrition, n (%)		0 (0.0)	0 (0.0)	0 (0.0)
At risk of malnutrition, n (%)		11 (7.0)	6 (4.4)	5 (23.8)	0.001
Well-nourished, n (%)		146 (93.0)	130 (95.6)	16 (76.2)	
BMI, [kg/m^2^], mean (SD)		26.5 (4.0)	26.6 (3.9)	25.9 (4.2)	0.468
Waist circumference [cm], Median (P25–P75)	Female n = 78	89.4 (81.2–99.2)	90.0 (82.4–99.4)	85.7 (76.6–97.9)	0.395
	Male n = 80	103.2 (94.7–109.1)	103.7 (94.7–109.1)	97.5 (93.3–111.8)	0.665
Leptin [mg/mL], median (P25-P75)		9.00 (4.34–17.67)	9.00 (4.41–34.3)	10.29 (3.71–17.99)	0.978
IL-6 [pg/mL], median (P25–P75)		2.91 (1.32–3.60)	2.93 (1.32–3.70)	2.89 (2.29–3.49)	0.971
TNF-α [pg/mL], median (P25–P75)		2.80 (1.18–4.03)	2.68 (1.18–3.97)	3.30 (2.92–4.63)	0.094

GDS, geriatric depression scale (0–15 points), a score below 5 points defines a non-depressive mood; MNA-LF, mini nutritional assessment-long form (0–30 Points), a score above 23.5 indicates being well-nourished; BMI, body mass index; IL-6: Interleukin 6; Tumor Necrosis Factor alpha (TNF-α); Comparison between the groups: chi-square test for categorical data, Mann-Whitney-U-test or *t*-test for continuous data

### Inflammation Status

**FIGURE 1:**
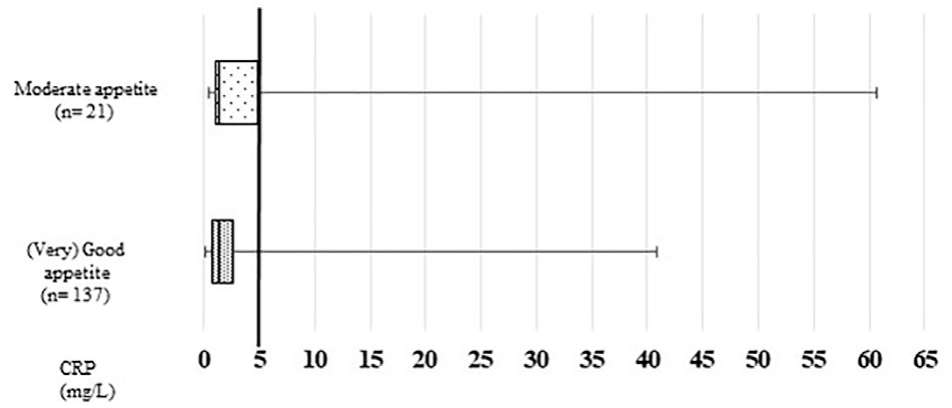
Participants’ CRP (C-reactive protein) levels according to appetite. Comparison between the groups, Mann-Whitney-U-test, *p* = 0.296.

Median CRP was 1.4 mg/L in the total sample and also in both appetite groups, however, IQR was broader in participants with moderate appetite (P25–P75: 1.0–4.9 mg/L) than those with a (very) good appetite (P25–P75: 0.8–2.6) (Figure 1). In total, 10.8% of participants had inflammation, 23.8% participants with moderate appetite compared to 8.8% participants with (very) good appetite (*p* = 0.038).

### Association Between Inflammation and Appetite

Table 2 shows the results of the regression analysis. In model 1, those with inflammation were 3.25 times more likely to have a moderate appetite, however with a broad CI: 1.01–10.44 (*p* = 0.047). In model 2, the association between inflammation and appetite was no longer significant, although the odds of having a moderate appetite among participants with inflammation was still about three times higher than in those with a good appetite. In model 3, GDS and leptin were significant confounders and the odds of having moderate appetite was 4.96 times higher in participants with inflammation, yet also with a broad CI (*p* = 0.028). In model 4, by considering other pro-inflammatory factor, GDS and leptin were still significant confounders, however, the association between inflammation and appetite was no longer significant.

**TABLE 2:** Association between inflammation (CRP ≥5.0 mg/L) and appetite (*n* = 158). Odds ratios (OR) and 95% confidence intervals (CI) for moderate appetite (vs. (very) good appetite)—unadjusted (model 1) and adjusted for potential confounders (models 2–4) (binary logistic regression).

		OR	95% CI	*p* value
Model 1	CRP	3.25	1.01–10.44	0.047
	constant	0.13		0.001
Model 2	CRP	3.06	0.90–10.40	0.074
	Age	1.13	0.96–1.33	0.148
	Sex	1.52	0.57–4.06	0.408
	constant	0.00		0.074
Model 3	CRP	4.96	1.18–20.75	0.028
	Age	1.10	0.91–1.34	0.328
	Sex	2.05	0.50–8.48	0.322
	Waist circumference	1.01	0.96–1.06	0.770
	Leptin	0.93	0.86–0.99	0.037
	GDS	1.94	1.35–2.81	0.001
	Number of medications	1.12	0.89–1.42	0.316
	constant	0.00		0.199
Model 4	CRP	3.78	0.77–18.58	0.102
	Age	1.12	0.91–1.37	0.292
	Sex	1.92	0.43–8.45	0.390
	Waist circumference	1.00	0.95–1.06	0.867
	Leptin	0.93	0.86–0.99	0.045
	GDS	2.01	1.36–2.97	0.001
	Number of medications	1.14	0.90–1.44	0.285
	TNF-α	1.37	0.98–1.92	0.062
	IL-6	0.97	0.66–1.43	0.882
	constant	0.00		0.156

OR, odds ratio; CI, confidence interval; CRP, c-reactive protein; GDS, geriatric depression scale

## Discussion

In this explorative cross-sectional analysis of data from very healthy, non-smoking community-dwelling older adults, inflammation was observed in nearly one quarter of participants with slightly impaired appetite. A significant association between inflammation and a moderate appetite has been observed unadjusted and adjusted for demographic and health confounders. Although in the fully adjusted model considering other inflammation markers in addition the significance disappeared, the odds of having moderate appetite were still more than 3-folds higher in those with inflammation than in those without, similar to the unadjusted model.

The majority of participants in our study reported a good or even very good appetite, which is in line with a study in community-dwelling older adults without functional impairment (mean age 74 years), where nearly 80% of individuals also had a very good or good appetite (van der Meij et al., 2017). However, in the aforementioned study around 20% reported an absence of a good appetite, including a moderate, poor, and very poor appetite (van der Meij et al., 2017), while in our study no one reported a poor or very poor appetite and only 13% had a moderate appetite, which can be explained by the fact that we recruited healthy older adults using strict eligibility criteria. For instance, older adults with BMI below 18.5 kg/m^2^ and weight loss of more than 5% in the previous 3 months were excluded.

In the two models considering GDS, emotional status emerged as asignificant covariate associated with even slightly reduced appetite, even though participants in both groups had no depressive mood. Similar to our findings, symptomatic depression was the strongest predictor of an impaired appetite in a study by Lee et al. In this study in more than 2000 well-functioning community-dwelling older adults with a mean age of 74 years, various factors (i.e., sociodemographic, health, medical, psychological, and sensory factors), which could have an association with an impaired appetite were evaluated by logistic regression. Appetite in the last month was assessed by a validated questionnaire (Lee et al., 2007). Nearly 12% of participants reported an impaired appetite, also in line with our findings. Further, in a recent study in 122 community-dwelling older adults (mean age 72 years), the prevalence of appetite loss, defined as a Simplified Nutritional Appetite Questionnaire (SNAQ) score of 14 points or lower, was significantly higher among those with depressive symptoms, defined as GDS ≥6 (Zukeran et al., 2021). Additionally in line with our findings, Zukeran et al. (2021) also found a significant association between the presence of depressive symptoms and appetite loss in a logistic regression model. Older adults with depressive mood may experience dysregulation of certain neurotransmitters such as serotonin which is involved in depression as well as eating regulation that could be a reason for a reduced appetite (de Boer et al., 2013).

Leptin, a cytokine-like peptide hormone, which inhibits hunger and decreases food intake (Morley, 2002), was a significant covariate, as well. Surprisingly, it was reversely associated with moderate appetite in our study. The circulating levels of leptin reflect the proportion of body adipose mass (Considine et al., 1996), although in our study older persons with BMI greater than 35 kg/m^2^ were excluded, participants with higher leptin levels had higher BMI (data are not shown). Thus, a possible explanation could be leptin resistance which is a reduction or a failure in the brain response to leptin, leading to a decline in leptin ability to suppress appetite (Crujeiras et al., 2015). Further, a very small number of participants with a (very) good appetite had inflammation as well as higher levels of leptin and these participants were indicated as outliers in the analyses (data are not shown). The probability of having leptin resistance might be the reason that these older persons, even though with inflammation, had a (very) good appetite and accordingly in model 3 it leads to the higher odds ratio for inflammation.

When the adjusted model was further controlled for other inflammation markers (TNF-α and IL-6), the association between CRP and appetite was no longer significant. However, TNF-α tended to be a significant factor of a moderate appetite, which is similar to the findings of Lee et al. (2007), where the inflammatory cytokines, IL-6 and TNF-α, were also among the factors addressed, and only TNF-α was significantly associated with an impaired appetite. Cytokines are released in response to chronic inflammation, and pro-Inflammatory cytokines, such as TNF-α, stimulate the hypothalamic corticotrophin releasing factor (CRF) as well, which acts as an anorectic agent (Morley, 2001). Moreover, IL-6 and TNF-α may be involved in reducing gastric motility and emptying, which consequently might reduce appetite and food intake (Yeh and Schuster, 1999). TNF-α stimulates the production of IL-6, and IL-6 can enhance the expression of CRP (Castell et al., 1990; Brüünsgaard and Pedersen, 2003). Besides, TNF-α independently of IL-6 can induce the synthesis of CRP (Bruunsgaard, 2006). The inflammatory cascade is very complex with a large number of components (Gabay and Kushner, 1999). In this study, we have chosen CRP as the main inflammation marker to examine its association with appetite in older adults, as it is one of the well-recognized markers of inflammation and is widely used as an indicator of inflammation state (Bruunsgaard, 2006).

Recently, in hospitalized older patients with a mean age above 80 years, an association between categorized CRP levels (<5 mg/L, 5–30 mg/L, and >30 mg/L), and appetite, evaluated by the single appetite question of SNAQ, was reported, whereas acute inflammation and chronic inflammatory were not related to appetite (Sieske et al., 2019). In contrast to robust participants in our study, however, this study examined very old and acutely ill patients with comorbidities, which may also affect appetite.

Appetite loss in older adults occurs due to several physiological, psychological and social changes (Morley and Thomas, 1999). Our results may indicate that inflammation might be an indicator for a reduced appetite, however, due to study design it is not possible to determine whether the association between inflammation and appetite is casual. Further, our finding should be interpreted with caution, as the small sample size and the small group size of only about 10% of participants with a reduced appetite as well as with inflammation resulted in a wide confidence interval and a low statistical power.

### Strengths and Limitations

The strength of our study is that the participants are a very homogenous group of healthy older adults and are well characterized. The main limitation of this study is the relatively small sample size. A further limitation is that appetite was assessed with a single question of how participants would estimate their appetite. A more comprehensive tool e.g., SNAQ (Wilson et al., 2005) might have been more suitable, however, there is also no gold standard assessment and appetite, in fact, is a very subjective feeling.

## Conclusion

In this cross-sectional analysis of data from very healthy community-dwelling older adults, we found hints for a potential inverse association between inflammation, measured as CRP, and a slightly reduced appetite. For a better understanding of the association between inflammation and appetite in healthy older people, studies in larger samples and desirably with a more comprehensive assessment of both inflammation and appetite are needed. Additionally, it would be worth investigating if other biomarkers of the immune system are also linked to a reduced appetite. Since there are several conditions, which can lead to an upregulation of inflammatory processes like chronic diseases, obesity, elevated oxidative stress or dysbiosis. Thus, it is of interest to investigate whether the association of inflammation with reduced appetite differs based on the underlying cause.

## Data Availability

The original contributions presented in the study are included in the article/Supplementary Material, further inquiries can be directed to the corresponding author.
